# Working with patients and the public to design an electronic health record interface: a qualitative mixed-methods study

**DOI:** 10.1186/s12911-019-0993-7

**Published:** 2019-12-03

**Authors:** Leigh R. Warren, Matthew Harrison, Sonal Arora, Ara Darzi

**Affiliations:** 1 Department of Surgery and Cancer, Patient Safety Translational Research Centre, St Mary’s Hospital Campus, Imperial College London, London, W21NY UK; 2 Helix Centre, St Mary’s Campus, Imperial College London, London, W21NY UK

**Keywords:** Electronic health records, Medical informatics, Healthcare service innovation, Patient safety, Clinical information systems

## Abstract

**Background:**

Enabling patients to be active users of their own medical records may promote the delivery of safe, efficient care across settings. Patients are rarely involved in designing digital health record systems which may make them unsuitable for patient use. We aimed to develop an evidence-based electronic health record (EHR) interface and participatory design process by involving patients and the public.

**Methods:**

Participants were recruited to multi-step workshops involving individual and group design activities. A mixture of quantitative and qualitative questionnaires and observational methods were used to collect participant perspectives on interface design and feedback on the workshop design process.

**Results:**

48 recruited participants identified several design principles and components of a patient-centred electronic medical record interface. Most participants indicated that an interactive timeline would be an appropriate way to depict a medical history. Several key principles and design components, including the use of specific colours and shapes for clinical events, were identified. Participants found the workshop design process utilised to be useful, interesting, enjoyable and beneficial to their understanding of the challenges of information exchange in healthcare.

**Conclusion:**

Patients and the public should be involved in EHR interface design if these systems are to be suitable for use by patient-users. Workshops, as used in this study, can provide an engaging format for patient design input. Design principles and components highlighted in this study should be considered when patient-facing EHR design interfaces are being developed.

## Background

Digital health technologies, including Electronic Health Records (EHRs), are increasingly used across all levels of healthcare [1]. Many digital systems are expanding to incorporate patient portals, patient reported outcomes, patient generated data, and social determinants of health [2–6]. Patient engagement with health records may have several benefits including patient satisfaction and better monitoring and management of chronic diseases, where care coordination is complex and frequently requires patient and carer engagement [5, 7, 8]. Previous attempts to help patients to take a more active role their own care through EHR portals have been limited due to a lack of accessibility, functionality, interoperability [9] and perceived dehumanisation of patients and their story associated with these electronic systems [10, 11]. If patient-accessible records are to succeed in improving patient-centred care, a human-factors derived, data-driven approach to new systems or improvements is required. Improved patient-facing interfaces using graphic representation of healthcare information is a clear mechanism to improve the utility of these records [12].

There are several examples of participatory design and patient and public involvement and engagement being successfully used to guide clinical service improvement and healthcare technology design [13–16]. These approaches attempt to involve key stakeholders in the design process to ensure that the output meets their needs and is usable [13]. Specific design and research methodologies to implement participatory design principles are not well documented in the medical and informatics literature.

This work aimed to develop an evidence-based electronic medical record interface and design process by involving and engaging patients and the public. Through structured workshops we aimed to provide a collaborative setting to guide the design of a digital interface to improve patient engagement with their medical records.

## Material and methods

### Design

This study used a mixed-method design incorporating questionnaires and observational methods within the framework of a workshop. An initial workshop pilot study was followed by the delivery of two multi-step workshops.

The workshop structure was used to provide a systematic framework for the research and design methods selected. The term ‘workshop’ generally refers to a process of creative group problem-solving [17]. The application of workshops as a research methodology therefore carries dual purposes of fulfilling participants’ expectations to achieve something related to their own interests, while producing reliable and valid data about the domain in question [18].

Principles of participatory design were applied throughout the workshops. Participatory design focuses on allowing the user to be a legitimate and acknowledged participant in the design process [19] and gives voices to those who are not in positions of power and decision making [20]. Participatory design has been shown to be a productive research tool to guide solutions to patient problems and clinical processes [21–23].

### Setting

Two back-to-back, identical workshops involving different participants were conducted. The duration of each workshop was 90 min. Three facilitators, all with some previous experience in facilitating group forums and/or participatory design workshops coordinated the sessions. One facilitator was a practicing clinician with a research interest in patient safety and digital health (LW). Two facilitators (including MH) had a background in healthcare graphic design and health technology development.

### Participant recruitment

To achieve the aims of this project, a sample of the general public was deemed the most appropriate and accessible target group. We aimed to recruit a broad range of ages and backgrounds, whilst ensuring an accessible method of recruitment for potential participants. To limit barriers to recruitment and to conform with our institutional approval for this project, we did not request patients to provide their own personal medical histories or active health issues during the study. Participants were recruited through advertisement of the session on a publicly accessible website, links to which were disseminated through social media applications. Limited spaces were also made available for walk-in participants on the day of the workshop. Registration for the workshop was conducted on a first-in, first-served basis. Capacity was limited to 25 participants per session. Participation in the workshop was voluntary and unpaid.

### Procedure

Design of the workshop procedure was based on participatory design principles [24, 25] and previously validated workshop methods including the use of case vignettes and step-by-step workshop methods published in the literature [14, 15]. An initial pilot study was conducted with four participants recruited via opportunistic sampling to trial and validate the workshop procedure. Feedback from the pilot study assisted in guiding the structure and content of the formal workshops including additional contextual information during the first step and additional time to complete design activities.

**Fig. 1:**
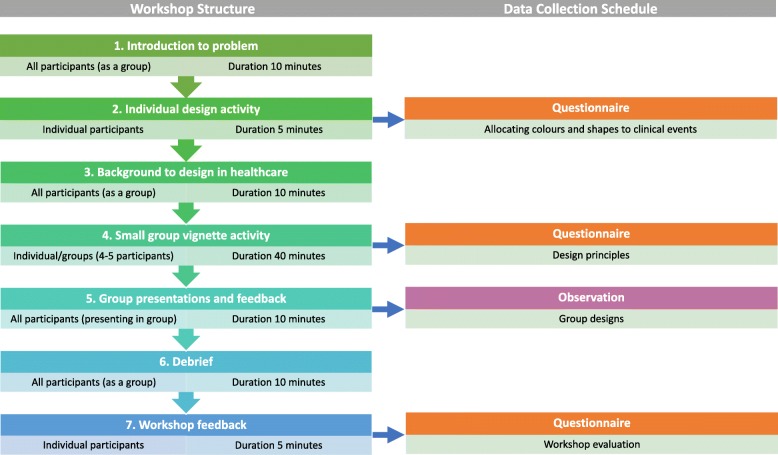
Steps of workshop procedure and data collection schedule, including composition of groups, duration of activity and data collection methods

Two identically structured workshop sessions followed a seven-step procedure illustrated in Fig. 1 and outlined in further detail below:

### Introduction to problem

An initial introduction to the workshop and evidence-based review of existing problems with electronic health records and health information exchange during patient transitions of care (TOC) was provided. Participants were shown examples of existing digital medical record interfaces and encouraged to consider how the design of these might be improved for patients. An overview of the workshop procedure and objectives was provided along with the opportunity for questions from participants.

### Individual design activity

Workshop participants were invited to complete two questionnaires relating to design in healthcare. Questionnaires related to A) associations between colours and common clinical events and B) associations between shapes and common clinical events. Each clinical event could be matched to only one colour or shape. Each colour or shape could be used more than once. Questionnaires were completed anonymously and individually.

### Background to design in healthcare

A presentation on the role and importance of design in healthcare was provided to participants. This was a didactic presentation that provided examples of innovative and successful design approaches that addressed healthcare problems, including transitions of care.

### Small group vignette activity

This interactive segment of the workshop required participants to work in small groups of 4 or 5 to develop a prototype interface design to graphically depict a patient medical history. One of five fictional clinical vignettes, each containing approximately 10 clinical events, were provided to participants. Each participant was initially provided with an open-ended questionnaire that related to the graphical depiction of a patient medical history. The 4 questions within this questionnaire covered perspectives on design principles, design structure and design elements including graphics and colours. These questionnaires were completed independently prior to the group work. In groups, designs were then produced using cardboard cut-outs and coloured pens on paper to depict the interface of a computer, tablet or smartphone device.

### Group presentation and feedback

Following the group task, participants nominated a group member to present the group prototype to the researchers and other workshop participants. They were asked to describe the overall structure of the design and specific details that they felt would benefit communication of the data provided with a potential user. Observation of interface design prototypes was conducted retrospectively using photographs of all designs which were independently analysed and categorised by two researchers.

### Debrief

This short segment facilitated an interactive group discussion regarding the tasks performed and overall perspectives on the workshop session within the setting of an open forum.

### Workshop feedback and evaluation

Individual evaluation of the workshop was undertaken by participants using a structured questionnaire. Questions covered the workshop experience and participant perspectives on the utility of the workshop to addressing the problem presented in step 1. Evaluation questionnaire forms were confidential and anonymous.

### Instruments

Colour and shape association questionnaires (step 2) used a closed-ended category-matching format and the design principles questionnaire used an open-ended format with 4 questions. The clinical events used in the questionnaires were developed using a process of identification and refinement through consensus with 2 active clinicians with over 5 years of healthcare experience (including LW). Vignettes were also developed by 2 clinicians and each aimed to capture a range of common clinical events including new diagnoses, investigations and interventions for each fictitious patient. These were written as a text block to minimise seeding of design structure. Verbal design briefs were provided by a clinician (LW) and healthcare graphic designer (MH) and aimed to be broad to limit bias. Evaluation questionnaires used in step 7 included 12 multi-point Likert scale questions.

### Outcomes

The primary outcome for this study was interface design principles and components for digital medical records. Patient and public perspectives on, and satisfaction with, the workshop procedure as an educational tool and participatory design process was also evaluated.

### Data analysis

Ordinal data from Likert scales was collated and analysed using Microsoft Excel. Qualitative content analysis from open-ended questionnaire sections was analysed using a Framework Method [26]. Content was initially reviewed line-by-line by two researchers independently. An inductive approach using open coding and development of an analytical framework preceded the generation of emerging themes. Themes were then refined, discussed between researchers to reach consensus and interpreted for discussion.

Retrospective observational analysis of the group task output was restricted to overall themes due to the complexity of content and to minimise the impact of bias. Themes for designs were allocated based on the thematic analysis of designs generated during the open-ended individual questionnaire responses during step 4. Using these themes, group prototype designs were classified into one of three categories; a) timeline design, b) anatomical design, c) other design. Analysis of photographs of interface designs was undertaken independently by two members of the research team (LW, MH).

## Results

### Clinical event colour association

**Fig. 2:**
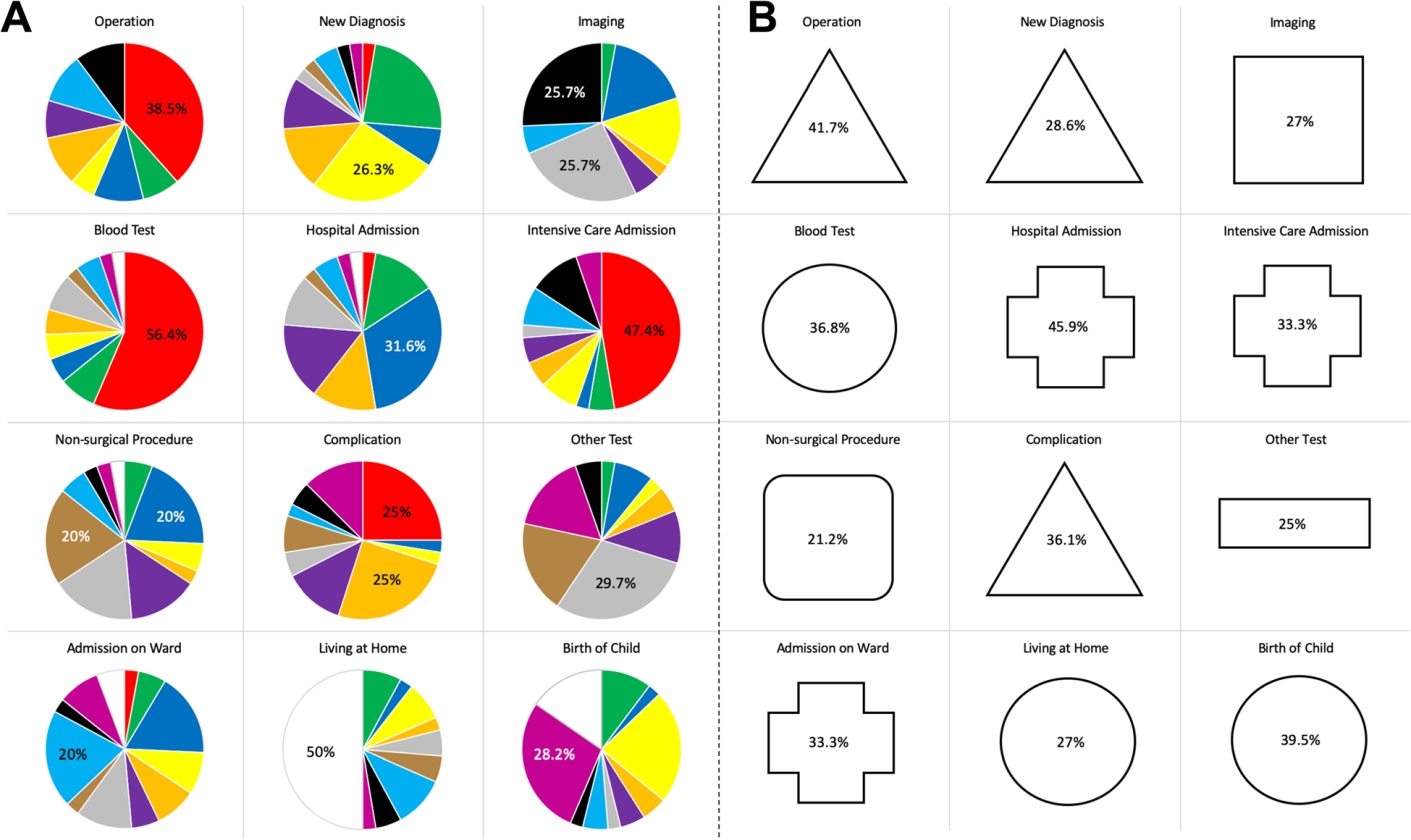
Association of colours (**a**) and shapes (**b**) to clinical events and relative frequency of principal choice(s)

A total of 48 participants were recruited and attended the workshop sessions. 40 participants undertook the task requiring the matching of 12 clinical events with 12 colours. There was consensus between participants for a principal colour match for several clinical events, as illustrated in (Fig. 2a). 56.4% (22/39) of participants indicated that a blood test should be graphically represented by the colour red, 50% (19/38) indicated that ‘living at home’ was appropriately indicated by white and 47.4% (18/38) that red was the best colour to associate with an Intensive Care Unit admission. Imaging was felt to be best represented by black (25.7% (9/35)) or grey (25.7% (9/35)).

### Clinical event shape association

40 participants undertook the task requiring the matching of 12 clinical events with 9 shapes. Participants independently agreed on a principal shape match as illustrated in (Fig. 2b) for several clinical events. Of the presented shapes, 45.9% (17/37) of participants felt that a hospital admission was most appropriately depicted with a cross, 41.7% (15/36) allocated a triangle to an operation and 39.5% (15/38) a circle to the birth of a child.

### Design principles

Several themes relating to the design of a digital transitions of care tool were identified following coding and thematic analysis of responses to the design principles questionnaire. Three key themes were agreed upon through consensus, each with several sub-themes allocated. Key themes identified were:

#### Overall design principles

Several participants noted that an interface design should focus on simplicity, clarity, beauty and user-friendliness. They felt that a design should be intuitive, engaging and enjoyable to use. They emphasised the importance of accuracy of information, the ability to rapidly assess information and the need to incorporate the diversity of disease. Some participants proposed that there should be a ‘hierarchy of information’ where emphasis is placed on more important, severe or relevant medical problems.

#### Design structure

Most participants commented that the structure of an interface design should be simple. Several overarching design structures emerged including a timeline design, anatomical-based design, calendar design and more abstract designs such as a cabinet with drawers corresponding to different medical problems.

#### Design elements

Participants identified the utility of interactive features and the use of infographics. Emphasis was placed on the use of colours, in particular the use of bright, primary colours and the use of colour to represent disease status, severity or corresponding medical specialty. Some participants suggested the use of warm colours such as red, orange and yellow to reflect severe problems and blue or green for a normal, non-active state. The use of either geometric or organic shapes was suggested as an important interface design element.

### Group vignette activity

10 small groups of 4 or 5 participants generated ten prototype interface designs through the small group vignette tasks. There was agreement between researchers on the categorisation of all designs. 6 (60%) prototypes were identified as using a timeline-based design, 2 (20%) prototypes used an anatomy-based design and 2 (20%) prototypes used other designs.

### Workshop evaluation

**Fig. 3:**
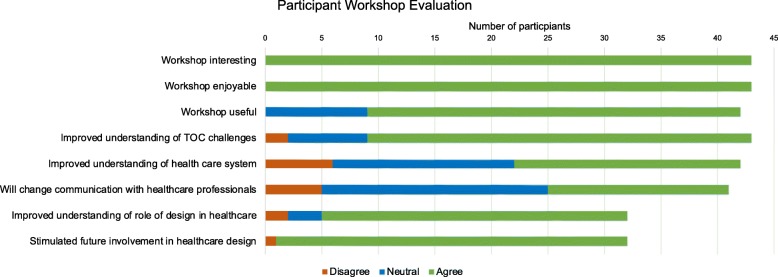
Participant evaluation of workshop from a 3-point Likert-type scale (Disagree, Neutral, Agree)

Quantitative evaluation questionnaires were undertaken by 43 participants. Non-responses to individual questions were excluded from analysis. Outcomes from the Likert-type scales are shown in Fig. 3. All responding participants found the workshop interesting and enjoyable. 78.6% (33/42) of participants felt that the workshop was useful and 79.1% (34/43) reported that it improved their understanding of the challenges of health information exchange during transitions of care. 47.6% (20/42) of participants felt that the workshop improved their understanding of the health care system and 39% (16/41) indicated that they will change their communication with healthcare professionals following the workshop.

### Development of a prototype interface using workshop output

**Fig. 4:**
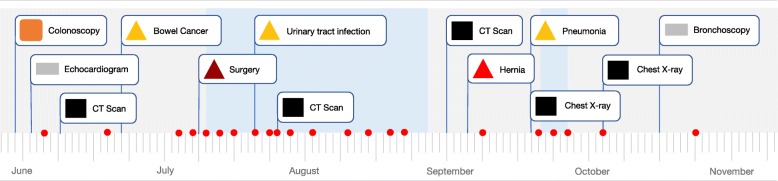
Prototype EHR interface design based on input from workshop participants

Following completion of the workshops, the research team developed a prototype EHR interface design using the key design principles identified in the workshops. This prototype design is shown in Fig. 4. The shapes and colours that workshop participants felt best reflected particular clinical events were used within the prototype design. Where workshop participants had selected the same colours for different clinical events, such as operations and complications, different shades of these colours were used in the prototype design. An interactive, digital version of this interface has been developed and will be compared with existing EHR interfaces currently in use.

## Discussion

This report describes the methods and outcomes of a novel approach to obtaining patient and public perspectives on the design of a digital medical record interface. Through the provision of two structured workshops, several design principles and components that are important to healthcare users were identified and a prototype design was developed.

### Design principles and components for digital medical records

The identification of design principles and components in this work complements and expands upon previous experimental work on patient portals [27]. The use of a timeline to graphically depict a patient history was identified as a suitable structural design element. This finding may indicate an inclination for patients to consider their medical history chronologically, which may differ from provider approaches which may often be system or disease-based [28]. Participants identified that digital medical record interfaces should be clear, simple, intuitive and include the use of colours and geometric graphics. Findings from questionnaires in this study indicate that there is consensus between patients on the use of specific colours or shapes to depict particular clinical events. Specific design elements identified, such as the use of a red circle to indicate a previous or upcoming blood test, may provide guidance for interface design across multiple health design applications. The use of colours and geometric shapes that were identified as suitable to indicate several different healthcare events, such as the colour red and circles, triangles or crosses, may require further user input to minimise overlap between these elements in interfaces. Alternatively, the use of different shades of the colours may be used, as was done in the prototype interface developed by our group shown in Fig. 4. Further work in this area to incorporate the design perspectives of other stakeholders such as providers and researchers would be beneficial and is a focus of future work for our group. We intend to use data from these studies to further guide, develop and test the prototype interface. The outcomes of comparisons of these interfaces with existing vendor interfaces will be addressed separately to this report.

### Improving healthcare quality and safety through better design

Healthcare is a high-risk industry and unsuitable design may lead to errors [29]. There are several examples of the use of graphic design to improve patient safety such as the design of tobacco packaging and public health information [30, 31]. New health-related digital technologies should be appropriately researched, designed and evaluated to reflect this [32]. Often, the important role of patients and the public in evaluating and redesigning care processes and digital systems has been neglected [6]. This work has demonstrated that novel and engaging methods of involving patients and the public can be both enjoyable for participants and constructive in their output.

There are several potential benefits of increased patient engagement with medical records, including an improved understanding of medical conditions and better preparation for visits with providers [2]. Interfaces that appeal to both patients and providers may act as a ‘boundary object’ to bridge functional knowledge and power gaps across different stakeholder positions during transitions of care [33]. Use of the design elements identified in this work may aid comprehension and synthesis of healthcare events, allowing patients to be more engaged with their healthcare journey and therefore improve safety during transitions of care.

### Workshop as a research and design process

The use of a workshop as a research and design tool has both benefits and limitations. Workshops follow a pre-defined, though not predictable, purpose [18] that allows participants to be involved in a process that achieves a goal. There are several examples of workshops being used in health service improvement and technology design [14–16]. Responses from the evaluation questionnaire indicated that participants found the workshop process used for this project to be enjoyable, useful and interesting. Participants indicated that this workshop stimulated their interest in being involved in future healthcare design work, further emphasising the potential value of the methods described in healthcare research and development. The research methods incorporated within the workshop generated valuable data to guide future healthcare research and design. The participatory design approaches used in this study could be used to better include patients and the public in the design of digital health technologies. The seven-step workshop framework described may be adapted and applied to other patient safety and healthcare issues.

### Limitations

The recruitment process used did not deliberately identify particular medical pathologies or medical histories. Similarly, we did not select or seek information relating to participant backgrounds or levels of health and information technology literacy. This approach was used to ensure that recruitment was simple and achieved sufficient participant numbers and to comply with personal data regulations and institutional approval for this study. Further studies looking at perspectives from more homogeneous patient subsets with differing healthcare experiences may enable targeting of design components for specific patient groups. This study used validated research methods within a novel workshop framework. The educational components within the workshop were structured to limit the introduction of bias to the research components. Some workshop participants did not partake in the questionnaire which reduced the yield of results. The closed-ended colour and shape-matching questionnaires were conducted prior to the design principles questionnaire and group tasks. This may have exposed participants to potential seeding and bias in subsequent responses including the use of colour and shapes in designs. Direct comparison of the developed prototype interface with existing EHR interfaces was not conducted in this study but is a future focus for our research group.

## Conclusion

The involvement and engagement of patients and the public is critical in ensuring that the design of digital technologies is patient-centred. Specific design principles and elements identified in this study should be considered when developing patient-facing digital medical record interfaces. This study has shown that patients value the role that they have to play in improving the quality and safety of healthcare technology and processes. Structured workshops can provide a suitable and enjoyable methodology for research and design involving patients and the public. Further research to expand and develop both the frequency and quality of patient input into health technology design are warranted.

## Data Availability

The datasets and materials used and/or analysed during the current study are available from the corresponding author on reasonable request.

## Abbreviations

EHR: Electronic Health Record
PPI: Patient and Public Involvement
TOC: Transition of Care
